# Reclaiming physician identity: It’s time to integrate ‘Doctor as Person’ into the CanMEDS framework 

**DOI:** 10.36834/cmej.69182

**Published:** 2020-08-06

**Authors:** J. Damon Dagnone, Susan Glover Takahashi, Cynthia R. Whitehead, Salvatore M. Spadafora

**Affiliations:** 1 Queen’s University, Ontario, Canada; 2 University of Toronto, Ontario, Canada

In 1996, the Royal College of Physicians & Surgeons of Canada adopted the CanMEDS framework with seven key roles: communicator, collaborator, health advocate, medical expert, manager, professional, and scholar.^1^ For many years, CanMEDS in various iterations has been globally recognized for identifying what patients need from their physicians. Like any model however, it is imperfect. For Canadian medical education to meet today’s challenges of providing care in constantly changing and overly-stressed healthcare settings, educators will need to help new physicians find ways to manage expectations and needs, while retaining their wellness and humanity.

Studies on burnout among physicians and other healthcare providers repeatedly tell a stark tale.^2^^,^^3^^,^^4^ A widely publicized survey conducted by the Canadian Medical Association in 2018 found high rates of burnout, depression, and suicidal ideation among physicians, with increased rates among women and physicians in their first five years in practice.^5^ Although survey respondents indicated that they were aware of physician health services, they expressed reluctance to access them as they thought their problems were not severe enough or they were ashamed to initiate access.

In its current formulation, the CanMEDS Framework has insufficient emphasis on the physician as a person. Given the magnitude of the problem of physician wellness, this is a disturbing deficiency. It is essential that the CanMEDS theoretical model be better informed by the mounting evidence that exists on wellness, healthcare systems, and dyscompetence.

Historically, we can identify how changing healthcare structures have contributed to these problems. In *Let me Heal*, historian Kenneth Ludmerer eloquently describes shifts in health care environments such that demands for efficiencies, shortened lengths of hospital stays, and increased standardization have shifted the provision of care to protocol, guideline and algorithm-driven approaches at the expense of reflective inquiry and patient-centeredness.^6^ With these new practice realities, we shouldn’t be surprised that it is a struggle for physicians to retain their sense of efficacy to provide holistic relational care to their patients.

Within our training milieus, the arrival of competency-based medical education has also presented challenges. Recent publications and conversations have used the concept of ‘epidemiology of competence’ to understand the risks and supports to competence for health professionals across the lifespan of their career (i.e. field-based education, residency or graduate medical education, and practice).^7^^,^^8^^,^^9^ This research suggests that maintaining competence requires healthy habits across personal, professional and practice environments to enable safe, effective and ethical practice. The current CanMEDs framework is insufficient to inform individuals, teams or organizations in the management or mitigation of efforts at the individual or systems level.^10^^,^^11^ If the CanMEDS framework were expanded to include the role of “Person”, the practice model would better align with evidence related to competence, dyscompetence management, and mitigation options.

CanMEDS is a successful and globally recognized competency framework that is used by numerous countries across five continents around the world as the basis for training physicians. It serves as a collective physician identity.^12^ Historically, the CanMEDS Framework was commissioned by specialist physicians in Canada in 1990, leveraging the work of the Educating Future Physicians of Ontario (EFPO) project that brought together medical schools in Ontario (Phase 1) to determine what the public expected of physicians.^13^ A broad range of groups including the disabled, multi-cultural groups, women, the elderly, and most health professions were consulted. Phase 1 of the project focused heavily on the roles of the physician, hereafter known as the EFPO Roles: medical expert, communicator, collaborator, gatekeeper, learner, health advocate, scientist, and person. In 1996, the Royal College of Physicians and Surgeons of Canada adopted seven of these roles into the CanMEDS framework, with revisions in 2005 & 2015, and the College of Family Physicians of Canada adapted these roles into CanMEDS FM in 2009.^1^^,^^12^

The ‘removal’ of the 8^th^ role: Physician as Person, has not been widely discussed. Originally, it was defined as one who is able to balance professional and personal roles and cope with the stress of professional demands. It also included sharing of oneself with patients to build trust. The framework urged educators to address in and through the curriculum the influence of professional demands on personal life and identify coping strategies.^13^ Despite broad support for inclusion of the Physician as Person role to address balance between professional and personal life, and as a person who enters relationships with patients, it was nevertheless absent from the CanMEDS framework launched in 1996 and the updates in 2005 and 2015.^14^ Thus, a vitally important aspect of sustainable practice was omitted from the framework that educators, learners, and practitioners use as scaffolding for physician identity, curricular planning and implementation. The Person role needs to be ‘found’ again to focus attention on the importance of self and self-care in objectives, learning activities and assessment of learners. Perhaps the Person role was less compelling in the 1990s. It is sorely needed now given the dilemma the profession finds itself in today. Given the health of practitioners the need for empathy for self, colleagues and patients, and now in light of the global COVID-19 public health crisis.

Adding both Doctor as Person to the CanMEDS framework and new curricular elements to undergraduate medication education (UGME) and postgraduate medical (PGME) training alone is not the answer. What is needed are concrete elements of implementation that span the continuum of medical training and practice. Medical schools, hospitals, clinics, and other healthcare institutions must create the time, space, and habits during workday hours to realize the intended impact of this initiative for physicians and all other interprofessional staff. Leveraging implementation science and the principles of change leadership, meaningful adoption likely requires “on the ground” innovations in UGME, PGME, and most importantly front-line physicians through continuing professional development (CPD). Examples include interprofessional hospital lunchtime forums such as “Vulnerability Rounds,” reflection-focused CPD experiences, career peer to peer mentors, and cross-appointments with teaching from the humanities disciplines. We must embrace opportunities for holistic discussions, exploration, debriefing, and reflections about personal and professional experiences including challenges, resources and supports.

Moving forward, the CanMEDS framework needs to be optimized by explicitly identifying who physicians are as people, better identifying the competencies required to navigate the personal and professional challenges we’re facing, and warding off the threat that exists in losing authentic human to human care interactions. An action-oriented approach that engages practicing physicians across the country, focuses on deeds instead of words alone, and works towards a shared understanding with data-informed research are some of the challenges ahead. We also wonder if we should visualize “Doctor as Person” as the stem that holds the CanMEDS flower up and which provides nourishment to the petals that have helped define our collective identity for more than twenty years. It is time to give the stem the recognition it deserves.
